# Effect of Stress Hyperglycemia on Neurological Deficit and Mortality in the Acute Ischemic Stroke People With and Without Diabetes

**DOI:** 10.3389/fneur.2020.576895

**Published:** 2020-09-24

**Authors:** Jiejie Li, Kehua Quan, Yilong Wang, Xingquan Zhao, Zixiao Li, Yuesong Pan, Hao Li, Liping Liu, Yongjun Wang

**Affiliations:** ^1^Department of Neurology, Beijing Tiantan Hospital, Capital Medical University, Beijing, China; ^2^China National Clinical Research Center for Neurological Diseases, Beijing, China; ^3^Center of Stroke, Beijing Institute for Brain Disorders, Beijing, China; ^4^Beijing Key Laboratory of Translational Medicine for Cerebrovascular Disease, Beijing, China

**Keywords:** stroke, glucose, glycated hemoglobin A, prognosis, China

## Abstract

**Objective:** To determine the relationship between stress hyperglycemia and prognosis of acute ischemic stroke people with and without diabetes.

**Methods:** A subgroup of 8,622 acute ischemic stroke people with baseline levels of fasting blood glucose and HbA1c from the China National Stroke Registry II were analyzed. Stress hyperglycemia was measured by glucose/glycated hemoglobin (HbA1c) ratio, calculated by fasting blood glucose divided by HbA1c. Diabetes was diagnosed according to medical history or a HbA1c level of ≥6.5%. The outcomes included severe neurological deficit defined as modified Rankin Scale score of 3 to 5 and all-cause death at 1 year. The associations between glucose/HbA1c ratio and neurological deficit and all-cause death were analyzed via logistic regression model and Cox proportional-hazards model, respectively. Subgroup analyses of participants with or without diabetes were performed separately.

**Results:** Totally 1,189 (13.7%) participants had severe neurological deficit, and 678 (7.9%) died within 1 year. Compared with the lowest quartile, the highest quartiles of glucose/HbA1c ratio were associated with elevated risk of worse neurological deficit (20.1% vs. 13.0%; adjusted OR, 1.83; 95%CI, 1.31–2.54, *p* = 0.001), and mortality (12.1% vs. 6.6%; adjusted HR, 2.04; 95% CI, 1.47–2.83, *p* < 0.0001) after adjusted for potential covariates. The association of glucose/HbA1c ratio with neurological deficit remained in the participants with and without diabetes, while it was only significant in the participants without diabetes as for the outcome of mortality.

**Conclusions:** Stress hyperglycemia, measured by glucose/HbA1c ratio, was associated with increased risk of severe neurological deficit and mortality within 1 year in the acute ischemic stroke people.

## Introduction

Stress hyperglycemia is the relative transient increase in glucose when an acute stress such as stroke occurred (1). It is present in approximately half of people with acute ischemic stroke (2, 3). Some (3) but not all (4) prior studies indicated acute stress hyperglycemia predicted increased risk of mortality and poor functional outcome after ischemic stroke. Most previous studies defined stress hyperglycemia according to absolute glucose level, including the fasting and random glucose levels, without considering the background glucose concentration, although they were analyzed by status of diabetes (3, 4). Glycated hemoglobin (HbA1c) is a well-validated marker for glucose control over 2–3 months, reflecting the background glucose level before the event (5). Recent studies demonstrated that relative hyperglycemia, defined as glucose/HbA1c ratio or admission glucose divided by estimated average glucose derived from HbA1c, might be a more reliable method to measure the degree of stress hyperglycemia and a better predictor for outcomes of acute illness than absolute hyperglycemia (6–8). Our recent study showed that relative hyperglycemia of glucose/HbA1c ratio was related to mortality in the ischemic stroke people without diabetes (9). However, its association with neurological functional disability is still undefined. Furthermore, people with diabetes could also develop stress hyperglycemia, which has been overlooked in many previous studies (1). In this study, we therefore aimed to investigate the relationship between stress hyperglycemia, measured by glucose/HbA1c ratio, and outcomes of acute ischemic stroke people with and without diabetes in a large stroke registry study in China.

## Methods

The data that support the findings of this study are available from the corresponding author upon reasonable request.

### Study Design and Site Selection

The cohort from China National Stroke Registry II (CNSR II) was used in this study. As described previously (10), the CNSR II was the nationwide initiative to establish a reliable national stroke database for assessing the delivery of stroke care in clinical practice and to identify areas that needed further improvement compared with CNSR I in 2007 (11). The criteria for site selection in CNSR II were similar as CNSR I (11), briefly including: (1) having at least one stroke neurologist; (2) at least two hospitals included from each of the 31 provinces and municipalities in mainland China; (3) participating voluntarily; and (4) ability to perform research (11). A total of 219 hospitals participated in CNSR II. The study was approved by the central Institutional Review Board at Beijing Tiantan Hospital. All participants or their legal proxies provided signed informed consent before enrollment.

### Study Participants

**Figure 1:**
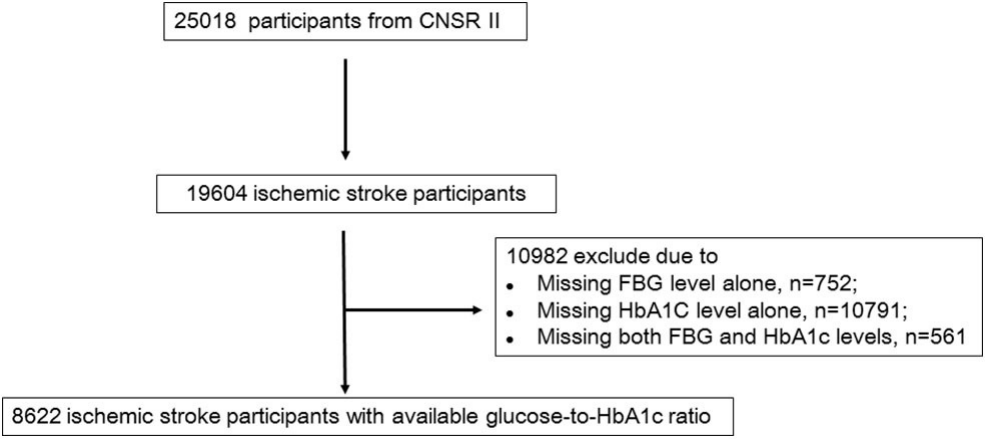
Flow chart showing the participant selection; CNSR, China National Stroke Registry; FBG, Fasting blood glucose.

Participants were consecutively recruited from centers from June 2012 to January 2013, if meeting the following criteria: (1) age>18 years; (2) diagnosis within 7 days of the index event, including ischemic stroke, TIA, spontaneous intracerebral hemorrhage, or subarachnoid hemorrhage; (3) direct hospital admission from the emergency department or physician's clinic; and (4) informed consent provided by the participant or legally authorized representative. Specifically, ischemic stroke was diagnosed according to the World Health Organization criteria combined with brain computed tomography (CT) or magnetic resonance imaging (MRI) confirmation (12). Of 25,018 participants with acute cerebrovascular events in CNSR II, 19,604 were diagnosed with acute ischemic stroke. Among these participants, 8,622 with baseline levels of fasting blood glucose and HbA1c were included (Figure 1 and Supplementary Table 1). Participants with diabetes were diagnosed according to a prior history of diabetes or a HbA1c level of ≥6.5% (13).

### Data Collection

Trained research coordinators at each hospital collected clinical data including demographics, detailed medical history, prestroke mRS, vascular risk factors, stroke severity, laboratory tests, treatments, complication, cerebrovascular events during hospitalization, and discharge status. Demographic variables included age and sex. The vascular risk factors included cigarette smoking, and medical history of hypertension, diabetes, hypercholesterolemia, ischemic stroke, TIA, myocardial infarction, heart failure and atrial fibrillation. Stroke severity was evaluated using National Institutes of Health Stroke Scale (NIHSS) score by physicians (14). Levels of fasting blood glucose, HbA1c, low-density lipoprotein cholesterol (LDL_C), triglyceride and high-sensitive C-reactive protein (hsCRP) on admission were extracted from medical records. All participants received routine stroke care according to their conditions.

### Follow-Up and Outcomes

The detailed follow-up procedure has been previously described (15). In brief, participants were followed up by telephone interview at 3, 6, and 12 months after stroke onset by trained research personals blinded to participant information, following standard scripts to collect information on stroke-related disability and all-cause death. Totally 960 (11.1%) patients were lost at 1 year. The outcomes included severe neurological deficit defined as modified Rankin Scale (mRS) score of 3–5 and all-cause death at 1 year.

### Statistical Analysis

Categorical variables were presented as percentages and continuous variables as medians with interquartile ranges. Baseline characteristics were analyzed by x^2^ statistics for the categorical variables and Kruskal-Wallis test for the continuous variables.

The glucose/HbA1c ratio was calculated by fasting blood glucose (mmol/L) divided by HbA1c (%) (6). The associations between glucose/HbA1c ratio and neurological deficit were analyzed by using logistic regression model, and the Cox proportional-hazards model was used for analyzing the association with mortality. Kaplan–Meier survival curve was applied to depict the occurrence of mortality and analyzed using the log-rank univariate test. Inflammatory response maybe a causative factor leading to high glucose level associated with poor outcomes in the patients with acute ischemic stroke, apart from stress. We therefore included demographic factors, prior published traditional or clinical risk factors and levels of hsCRP in the multivariate model. Hazard ratios (HRs) or Odd ratios (ORs) and their 95% confidence intervals (CIs) were calculated. A 2-sided *P* < 0.05 was considered to indicate statistical significance. SAS software, version 9.4 (SAS Institute, Inc., Cary, NC) was used for all statistical analyses.

## Result

### Participants Characteristics

Among 8,622 participants included in the final study, 2,398 (27.8%) had prior diabetes, 2984 (34.6%) had a HbA1c level of ≥6.5% and 3488 (40.5%) had either a history of diabetes or a HbA1c level of ≥6.5%. The medians of fasting blood glucose and HbA1c for all participants, participants with diabetes and participants without diabetes were 5.7 mmol/L (interquartile, 5.0–7.4 mmol/L) and 42.1 mmol/mol (6.0%) [interquartile, 36.6–53.0 mmol/mol (5.5–7.0%)], 7.8 mmol/L (interquartile, 6.1–10.3 mmol/L) and 58.5 mmol/mol (7.5%) [interquartile, 49.7–74.9 mmol/mol (6.7–9.0%)], and 5.2 mmol/L (interquartile, 4.7–5.8 mmol/L) and 37.7 mmol/mol (5.6%) [interquartile, 34.4–41.0 mmol/mol (5.3–5.9%)], respectively. The baseline characteristics of participants by quartiles of glucose/HbA1c ratio were shown in Table 1. Participants with higher glucose/HbA1c ratio were significantly younger, female, non-smokers, and had histories of hypertension and diabetes but not hypercholesterolemia and atrial fibrillation, higher baseline NIHSS and mRS scores, higher systolic/diastolic blood pressure, and higher levels of fasting blood glucose, HbA1c, LDL_C, triglyceride, and hsCRP (Table 1).

**Table 1:** Baseline characteristics of participants according to glucose/HbA1c ratio.

	**Q1 (*n* = 2,157)**	**Q2 (*n* = 2,153)**	**Q3 (*n* = 2,163)**	**Q4 (*n* = 2,149)**	***P*-value**
Age, years, median (IQR)	67 (58–75)	65 (57–75)	65 (56–74)	64 (55–74)	<0.001
Male, *n* (%)	1,426 (66.1)	1,334 (62.0)	1,386 (64.1)	1,269 (59.1)	<0.001
Current or previous smoking, *n* (%)	989 (45.9)	934 (43.4)	920 (42.5)	876 (40.8)	0.008
**Medical history**, ***n*** **(%)**					
Ischemic stroke	631 (29.3)	641 (29.8)	679 (31.4)	704 (32.8)	0.052
TIA	90 (4.2)	96 (4.5)	91 (4.2)	77 (3.6)	0.521
Hypertension	1,347 (62.5)	1,422 (66.1)	1,466 (67.8)	1,442 (67.1)	0.001
Diabetes	508 (23.6)	365 (17.0)	548 (25.3)	977 (45.5)	<0.001
Hypercholesterolemia	336 (15.6)	256 (11.9)	295 (13.6)	293 (13.6)	0.006
Myocardial infarction	51 (2.4)	39 (1.8)	45 (2.1)	56 (2.6)	0.316
Congestive heart failure	14 (0.7)	12 (0.6)	21 (1.0)	23 (1.1)	0.179
Atrial fibrillation	195 (9.0)	123 (5.7)	138 (6.4)	174 (8.1)	<0.001
Baseline NIHSS ≤ 3, n(%)	1,103 (51.1)	1,105 (51.3)	1,059 (49.0)	928 (43.2)	<0.001
Baseline mRS ≤ 2, *n* (%)	1,945 (90.2)	1,931 (89.7)	1,929 (89.2)	1,878 (87.4)	0.020
Baseline systolic blood pressure, mm Hg	146 (130–160)	150 (130–164)	150 (135–160)	150 (135–168)	<0.001
Baseline diastolic blood pressure, mm Hg	85 (80–92)	87 (80–97)	90 (80–96)	90 (80–100)	<0.001
**Laboratory data, median (IQR)**					
FBG, mmol/L	4.7 (4.3–5.2)	5.3 (4.9–5.8)	6.0 (5.5–7.0)	8.7 (7.0–11.7)	<0.0001
HBA1c, mmol/mol	42.1 (38.8–51.9)	39.9 (36.6–45.4)	39.9 (34.4–50.8)	48.6 (36.6–66.1)	<0.0001
HBA1c, %	6.0 (5.7–6.9)	5.8 (5.5–6.3)	5.8 (5.3–6.8)	6.6 (5.5–8.2)	<0.0001
LDL_C, mmol/L	2.7 (2.1–3.3)	2.7 (2.2–3.3)	2.8 (2.2–3.4)	2.9 (2.2–3.5)	<0.001
Triglyceride, mmol/L	1.3 (0.9–1.8)	1.3 (1.0–1.9)	1.4 (1.0–2.1)	1.6 (1.1–2.4)	<0.001
hsCRP, mg/L	2.0 (0.8–5.0)	2.5 (0.9–5.7)	2.6 (0.9–5.7)	3.0 (1.0–7.9)	<0.001

*IQR, interquartile range; TIA, transient ischemic stroke; NIHSS, National Institutes of Health Stroke Scale; mRS, modified Rankin Scale; FBG, fasting blood glucose; LDL_C, low-density lipoprotein cholesterol; HDL_C, high-density lipoprotein cholesterol; and hsCRP, high-sensitive C-reactive protein*.

*Glucose/HbA1c ratio was divided into 4 levels by quartiles as follows: quartile 1 (Q1), ≤15.30; quartile 2 (Q2),15.30–17.43; quartile 3 (Q3), 17.43–20.16; quartile 4 (Q4), >20.16*.

### Associations of Glucose/HbA1c Ratio With Outcomes

A total of 1189 (13.7%) participants had severe neurological deficit (mRS, 3–5), and 678 (7.9%) deaths occurred within 1 year. Compared with the lowest quartile, the third (19.0% vs. 13.0%; adjusted OR, 1.68; 95% CI, 1.24–2.26; *p* = 0.001) and highest (20.1% vs. 13.0%; adjusted OR, 1.83; 95% CI, 1.31–2.54, *p* = 0.001) quartiles of glucose/HbA1c ratio were independently associated with elevated risk of worse neurological deficit (Table 2).

**Table 2:** Associations of Glucose/HbA1c Ratio with Neurological Deficit and All-cause Death at 1 year.

**Participants**	**Outcomes**	**Glucose/HbA1c**	**Event, *n* (%)**	**HR/OR (95% CI)**	***P*-value**	**Adjusted HR/OR**	***P*-value**
		**Ratio^†^**				**(95% CI)^‡^**	
All	Neurological Deficit (n = 6,984)	Q1 (n = 1,760)	229 (13.0)	Reference	/	Reference	/
		Q2 (n = 1,799)	290 (16.1)	1.28 (1.07–1.55)	0.009	1.31 (0.97–1.77)	0.08
		Q3 (n = 1,775)	338 (19.0)	1.57 (1.31–1.89)	<0.0001	1.68 (1.24–2.26)	0.001
		Q4 (n = 1,650)	332 (20.1)	1.68 (1.40–2.02)	<0.0001	1.83 (1.31–2.54)	0.001
	Death (n = 8,622)	Q1 (n = 2,157)	142 (6.6)	Reference	/	Reference	/
		Q2 (n = 2,153)	126 (5.9)	0.88 (0.69–1.12)	0.30	0.76 (0.53–1.09)	0.14
		Q3 (n = 2,163)	150 (6.9)	1.06 (0.84–1.33)	0.63	1.04 (0.74–1.47)	0.81
		Q4 (n = 2,149)	260 (12.1)	1.92 (1.56–2.35)	<0.0001	2.04 (1.47–2.83)	<0.0001
With Diabetes	Neurological deficit (n = 2,838)	Q1 (n = 568)	99 (14.8)	Reference	/	Reference	/
		Q2 (n = 404)	93 (18.7)	1.32 (0.97–1.80)	0.08	1.51 (0.86–2.67)	0.15
		Q3 (n = 535)	120 (18.3)	1.29 (0.96–1.72)	0.09	1.71 (1.00–2.92)	0.05
		Q4 (n = 817)	202 (19.8)	1.42 (1.09–1.85)	0.009	1.69 (1.01–2.84)	0.05
	Death (n = 3,488)	Q1 (n = 828)	63 (7.6)	Reference	/	Reference	/
		Q2 (n = 579)	39 (6.7)	0.86 (0.57–1.28)	0.45	0.62 (0.32–1.20)	0.16
		Q3 (n = 786)	56 (7.1)	0.92 (0.64–1.32)	0.64	0.75 (0.41–1.38)	0.36
		Q4 (n = 1,295)	128 (9.9)	1.32 (0.98–1.79)	0.07	1.31 (0.78–2.19)	0.30
Without Diabetes	Neurological deficit (n = 4,146)	Q1 (n = 1,093)	130 (11.9)	Reference	/	Reference	/
		Q2 (n = 1,302)	197 (15.1)	1.32 (1.04–1.68)	0.02	1.26 (0.88–1.81)	0.21
		Q3 (n = 1,120)	218 (19.5)	1.79 (1.42–2.27)	<0.0001	1.69 (1.17–2.44)	0.005
		Q4 (n = 631)	130 (20.6)	1.92 (1.47–2.51)	<0.0001	2.28 (1.45–3.59)	0.0004
	Death (n = 5,134)	Q1 (n = 1,329)	79 (5.9)	Reference	/	Reference	/
		Q2 (n = 1,574)	87 (5.5)	0.93 (0.69–1.27)	0.66	0.83 (0.53–1.29)	0.41
		Q3 (n = 1,377)	94 (6.8)	1.17 (0.87–1.58)	0.30	1.18 (0.76–1.81)	0.46
		Q4 (n = 854)	132 (15.5)	2.78 (2.10–3.67)	<0.0001	2.81 (1.83–4.31)	<0.0001

*HR indicates hazard ratio; OR, odds ratio; and CI, confidence interval*.

† *Glucose/HbA1c ratio was divided into 4 levels by quartiles as follows: quartile 1 (Q1), ≤15.30; quartile 2 (Q2), 15.30–17.43; quartile 3 (Q3), 17.43–20.16; quartile 4 (Q4), >20.16*.

‡ *For all participants and those with diabetes, the models were adjusted for variables of age, sex, current, or previous smoking, medical histories of hypertension, diabetes, hypercholesterolemia, atrial fibrillation, transient ischemic stroke, ischemic stroke, myocardial infarction and congestive heart failure, baseline NIHSS and mRS scores, baseline systolic and diastolic blood pressures, baseline levels of low-density lipoprotein cholesterol, triglyceride and hsCRP; for the participants without diabetes, the models were adjusted for the aforementioned variables except for the history of diabetes*.

**Figure 2:**
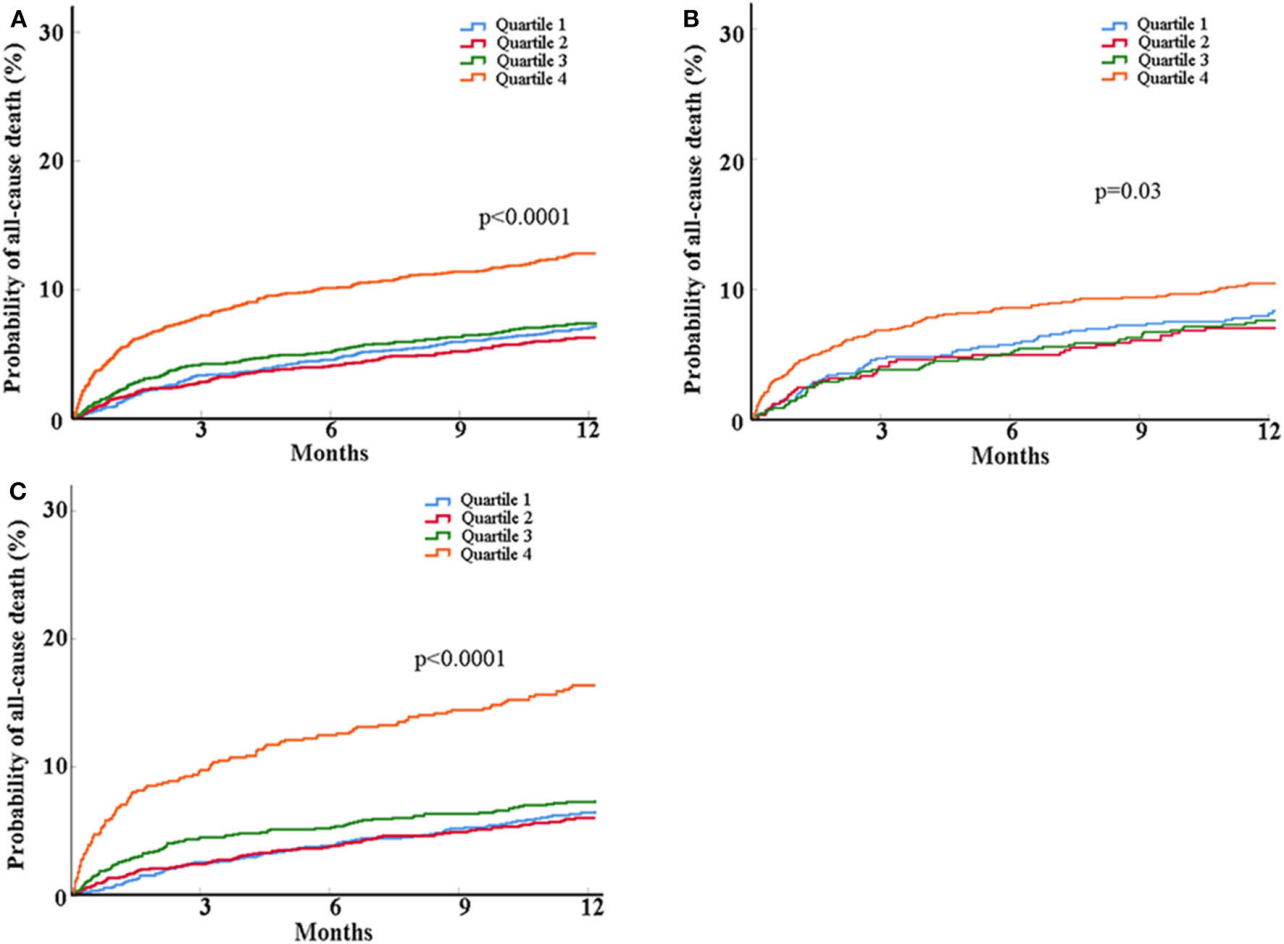
Kaplan-Meier curves for probability of all-cause death within 1 year in all patients **(A)** and the patients with **(B)** and without diabetes **(C)**. Patients were classified into four groups based on glucose/HbA1c ratio as follows: quartile 1 (Q1), ≤15.30; quartile 2 (Q2), 15.30–17.43; quartile 3 (Q3), 17.43–20.16; quartile 4 (Q4), >20.16. Log-rank tests were performed and the *p*-values were presented.

As for the outcome of mortality, participants in the highest quartiles of glucose/HbA1c ratio had around 2-fold of risk of mortality at 1 year than those in the lowest quartiles after fully adjusted for potential covariates (12.1% vs. 6.6%; adjusted HR, 2.04; 95% CI, 1.47–2.83, *p* < 0.0001) (Table 2 and Figure 2).

### Associations of Glucose/HbA1c Ratio With Outcomes in People With and Without Diabetes

When stratifying participants into those with and without diabetes, in the people with diabetes, the highest quartiles of glucose/HbA1c ratio was related to increased risk of poor neurological function recovery (adjusted OR, 1.69; 95% CI, 1.01–2.84, *p* = 0.047) but not mortality, compared with the lowest quartiles (Table 2 and Figure 2). In the people without diabetes, the associations of glucose/HbA1c ratio with neurological deficit (third vs. first quartiles of glucose/ HbA1c ratio: adjusted OR, 1.69; 95% CI, 1.17–2.44; *p* = 0.005; forth vs. first quartiles of glucose/HbA1c ratio: adjusted OR, 2.28; 95% CI, 1.45–3.59; *p* = 0.0004) and mortality (highest vs. lowest quartiles of glucose/HbA1c ratio: adjusted HR, 2.81; 95% CI, 1.83–4.31; *p* < 0.0001) were both significant in the crude and multivariate models (Table 2 and Figure 2).

## Discussion

The major finding of this study is that relative stress hyperglycemia measured by Glucose/HbA1c ratio independently predicts severe neurological deficit within 1 year in the acute ischemic stroke people, irrespective of diabetes status. And its association with mortality at 1 year is more apparent in those people without diagnosed or underlying diabetes.

There was controversy regarding the association between stress hyperglycemia and prognosis of ischemic stroke. An early meta-analysis found that acute stress hyperglycemia was associated with an increased risk of in-hospital mortality and poor function recovery in ischemic stroke people (3). Other studies subsequently showed that hyperglycemia predicted worse functional outcome at 6-month (16) and 1 year (17) in the people with acute ischemic stroke. And Roquer et al. also reported that hyperglycemia was related with 3-month mortality in acute ischemic stroke people without diabetes or with diabetes and previous good glycemic control, but not in those with diabetes and previous poor glycemic control (18). However, a recent prospective study did not demonstrate significant relationship between stress hyperglycemia and risk of in-hospital mortality and poor functional outcome after stroke (4). Difference of study population and definition of stress hyperglycemia might partly account for these discrepancies. Besides that, the discordance of included pertinent confounding factors, such as stroke severity, might also affect the predictive value of stress hyperglycemia (4, 19, 20). On the other hand, stress hyperglycemia was usually diagnosed according to absolute hyperglycemia, excluding prior diabetes or preexisting diabetes with deterioration of premorbid glycemic control, which in fact might also have stress hyperglycemia (1, 3). Furthermore, this definition failed to differentiate stress hyperglycemia from newly diagnosed or underlying albeit undiagnosed diabetes and did not consider the background glucose concentration. Unlike absolutely hyperglycemia, relative measures of hyperglycemia, such as glucose/ HbA1c ratio, reflected the relative acute rapid increase of blood glucose level compared with premorbid glucose status. Previous study showed that such relative measures controlling for background glycemia better predicted prognosis of critical illness than absolute hyperglycemia (6–8). We previously found that glucose/HbA1c ratio was related to all-cause death in people with acute ischemic stroke (9). In the current study, we further added evidence that glucose/HbA1c ratio could predict neurological deficit, in addition to mortality. In light of disability from stroke bringing huge personal and societal burden, early recognition and management of high-risk patients is of great importance. Blood markers are promising to aid in the risk stratification and individual treatment of ischemic stroke (21, 22). A line of evidence have shown that elevated blood glucose levels are associated with unfavorable outcome in the patients with ischemic stroke (3, 23–26). However, since hyperglycemia at the acute phage of ischemic stroke could be due to poor control of glucose in the diabetic patients, most prior studies excluded the diabetic patients (25, 26). People with diabetes could have stress hyperglycemia as well. Our study at least suggested a practical and economical measurement of stress hyperglycemia to predict the prognosis of stroke in the people with or without diabetes. Furthermore, taking background glucose into account has been emphasized in the current study base on the observation that for the patients with elevated fasting blood glucose, the prognosis might not be as poor as expected if accompany with high HbA1c. Our findings might shed some light on the individual glucose management in the acute ischemic stroke.

On the other hand, it has been observed that there is discordance in the associations of absolutely hyperglycemia with functional outcome and mortality between people with and without diabetes (3, 25, 26). Similarly, the relationship of higher glucose/HbA1c ratio with mortality was only apparent in the people without diabetes in our study. However, we did not find any difference in the predictive value of stress hyperglycemia in neurological function deficit between people with and without diabetes. The clear reasons for the discrepancy were still unknown. One possible explanation could be the difference in the definition of stress hyperglycemia as elaborated above. Secondarily, unlike majority of the previous studies, we diagnosed diabetes according to HbA1c levels in addition to prior history, being capable of differentiating people without diabetes from those with underlying but undiagnosed diabetes (25, 26). Thirdly, poor functional outcome was defined as a mRS score of >3 which included mortality in some study (25), which was different from ours as well.

Strengths of our study included a large sample size of ischemic stroke people with outcomes of interest such as functional disability and mortality, distinguishing people with underlying diabetes by using the measurement of HbA1c, as well as the inclusion of stroke severity when analyzing the association between stress hyperglycemia and outcomes. Nonetheless, some limitations should be noticed while interpreting our results. First, the major one was its retrospective design. Further randomized controlled trials are needed to confirm our findings. Second, participants with missing baseline fasting blood glucose or HbA1c levels were not included, so a selection bias might exist. Third, the infarct size was not available. Though it has been suggested that hyperglycemia contributed to the worsening of the initial infarct, irrespective of volume, and severity (25), debate continues as to if it is a contributing factor to the more severe stroke or merely a stress response to a larger infarct. Forth, the lack of exact cause of death impeded the understanding of the underlying causes for elevated glucose/HbA1c ratio. Fifth, as our cohort only comprised Chinese adult people with ischemic stroke, these results may not be generalizable to other races and ethnicities.

## Data Availability Statement

All datasets presented in this study are included in the article/Supplementary Material.

## Ethics Statement

The studies involving human participants were reviewed and approved by Central Institutional Review Board at Beijing Tiantan Hospital. The patients/participants provided their written informed consent to participate in this study.

## Author Contributions

JL and YoW conceptualized this work. YiW, XZ, ZL, HL, LL, and YoW supervised the study. JL, YP, and HL performed the statistical analysis. JL and KQ prepared the manuscript. YiW, XZ, ZL, and LL revised the manuscript. All authors approved the protocol.

## Conflict of Interest

The authors declare that the research was conducted in the absence of any commercial or financial relationships that could be construed as a potential conflict of interest.
