# Supplementary material for: Effect of Stress Hyperglycemia on Neurological Deficit and Mortality in the Acute Ischemic Stroke People With and Without Diabetes
Source: Front Neurol. 2020 Sep 24;11:576895. doi: 10.3389/fneur.2020.576895 (PMC7542306; doi:10.3389/fneur.2020.576895)
Supplement: Supplementary file 1 [file Table_1.DOCX]

SUPPLEMENTAL MATERIAL

Table I Demographic and Clinical Characteristics Between Included and Excluded Participants

|  | Participants Included in this substudy  n=8622 | Other Ischemic Stroke Participants in CNSR II  n=10982 | P value |
| --- | --- | --- | --- |
| Age, years, median (IQR) | 65 (57-75) | 65 (57-74) | 0.002 |
| Male, n(%) | 5415 (62.8) | 7022 (63.9) | 0.101 |
| Current or previous smoking, n(%) | 3719 (43.1) | 4953 (45.1) | 0.006 |
| Medical history, n(%) |  |  |  |
| Ischemic stroke | 2655 (30.8) | 3466 (31.6) | 0.250 |
| TIA | 354 (4.1) | 475 (4.3) | 0.449 |
| Hypertension | 5677 (65.8) | 7020 (63.9) | 0.005 |
| Diabetes mellitus | 2398 (27.8) | 1662 (15.1) | <0.001 |
| Hypercholesterolemia | 1180 (13.7) | 1190 (10.8) | <0.001 |
| Myocardia infarction | 191 (2.2) | 288 (2.6) | 0.067 |
| Congestive heart failure | 70 (0.8) | 97 (0.9) | 0.589 |
| Atrial fibrillation | 630 (7.3) | 752 (6.9) | 0.212 |
| Baseline NIHSS ≤3, n(%) | 4195 (48.7) | 4924 (44.8) | <0.001 |
| Baseline mRS ≤2, n(%) | 7683 (89.1) | 9909 (90.2) | 0.010 |

IQR, interquartile range; TIA, transient ischemic stroke; NIHSS, National Institutes of Health Stroke Scale; mRS, modified Rankin Scale.
